# Shutting Down Sensorimotor Interferences after Stroke: A Proof-of-Principle SMR Neurofeedback Study

**DOI:** 10.3389/fnhum.2016.00348

**Published:** 2016-07-15

**Authors:** Johanna L. Reichert, Silvia E. Kober, Daniela Schweiger, Peter Grieshofer, Christa Neuper, Guilherme Wood

**Affiliations:** ^1^Institute of Psychology, University of GrazGraz, Austria; ^2^BioTechMed GrazGraz, Austria; ^3^Klinik Judendorf-StraßengelGraz, Austria

**Keywords:** neurofeedback, sensorimotor rhythm, memory, aging, stroke

## Abstract

**Introduction:** Neurofeedback training aims at learning self-regulation of brain activity underlying cognitive, emotional or physiological functions. Despite of promising investigations on neurofeedback as a tool for cognitive rehabilitation in neurological diseases, such as after stroke, there is still a lack of research on feasibility and efficiency of neurofeedback in this field.

**Methods:** The present study aimed at investigating behavioral and electrophysiological effects of 10 sessions of sensorimotor rhythm (SMR) neurofeedback in a 74-years-old stroke patient (UG20). Based on previous results in healthy young participants, we hypothesized that SMR neurofeedback leads to a decrease in sensorimotor interferences and improved stimulus processing, reflected by changes in event-related potentials (ERPs) and electrophysiological coherence. To assess whether UG20 benefited from the training as much as healthy persons of a similar age, a healthy control group of *N* = 10 elderly persons was trained as well. Before and after neurofeedback training, participants took part in a multichannel electroencephalography measurement conducted during a non-verbal and a verbal learning task.

**Results:** Both UG20 and the healthy controls were able to regulate their SMR activity during neurofeedback training. Moreover, in a non-verbal learning task, changes in ERPs and coherence were observed after training: UG20 showed a better performance in the non-verbal learning task and a higher P3 amplitude after training than before, and coherence between central and parietal electrodes decreased after training. The control group also showed a behavioral improvement in the non-verbal learning task and tendencies for higher P3 amplitudes and decreased central-parietal coherence after training. Single-case analyses indicated that the changes observed in UG20 were not smaller than the changes in healthy controls.

**Conclusion:** Neurofeedback can be successfully applied in a stroke patient and in healthy elderly persons. We suggest that SMR neurofeedback leads to a shutting-down of sensorimotor interferences which benefits semantic encoding and retrieval.

## Introduction

Electroencephalography (EEG)-based neurofeedback is a promising tool for cognitive improvement and rehabilitation. While most traditional cognitive trainings consist of specific tasks that aim at improving cognitive functions, neurofeedback is aimed at directly regulating the brain activity underlying cognitive functioning. An important possible future area of application for neurofeedback is cognitive rehabilitation of neurological diseases, such as stroke (Doppelmayr et al., 2007; Hofer et al., 2014), as neurofeedback might be used to directly up-regulate certain aspects of brain activity while suppressing dysfunctional activations. During neurofeedback training, brain signals are recorded, processed and fed back to participants on a computer screen. In most cases, the feedback is presented auditorily or visually, for example as moving bars that have to be steered in a certain direction. This feedback enables participants to voluntarily control their own electrical brain activity.

During sensorimotor rhythm (SMR)-based neurofeedback, the power of SMR, a frequency band ranging from 12 to 15 Hz, is extracted from the EEG signal. SMR is an oscillatory rhythm recorded over central scalp regions, that is supposed to be originating from the thalamic nuclei, specifically from ventroposterior lateral and reticular nuclei (Sterman, 1996, 2000). SMR is observed when one is motionless but mentally focused and attentive and is suppressed when motor tasks or motor imagery are carried out (Pfurtscheller, 1981; Sterman, 1996). Since the beginnings of neurofeedback research, SMR has been utilized as a feedback frequency band. Several studies have shown that SMR neurofeedback training can lead to cognitive improvements, mainly in memory functions and attention (Vernon et al., 2003; Egner and Gruzelier, 2004; Hoedlmoser et al., 2008; Doppelmayr and Weber, 2011; Kober et al., 2015b). Underscoring its presumable effect of reducing brain excitability, SMR neurofeedback has been successfully applied in diseases such as epilepsy and ADHD (Lubar et al., 1995; Tinius and Tinius, 2000; Sterman and Egner, 2006). It was suggested that SMR might facilitate thalamic inhibitory mechanisms (Sterman, 1996, 2000; Egner and Gruzelier, 2004), and block motor activity that interferes with information processing (Pfurtscheller, 1992; Sterman, 1996). In accordance with this assumption, we found in a previous study that SMR-based neurofeedback training of healthy young adults leads to cognitive improvements related to changes in task-related electrophysiological parameters. Components of event-related potentials (ERPs), the N1 and P3, were increased after SMR training as compared to pre-training, indicating more intensive stimulus processing (Kober et al., 2015b). Importantly, functional brain connectivity between motor areas and visual processing areas was reduced after SMR training, while performance in a verbal memory task was improved. Thus, these results support the idea that SMR up-training leads to an enhanced blocking of sensorimotor interference, which might be responsible for the observed improvements in stimulus processing and memory function (Kober et al., 2015b). However, these results were obtained in a healthy, young sample and studies assessing the functionality of SMR neurofeedback in elderly persons and in persons suffering from neurological diseases, such as stroke (Doppelmayr et al., 2007; Hofer et al., 2014), are still scarce. Therefore, we aimed to investigate the effects of SMR neurofeedback in a single-case of stroke. Around 32% of stroke survivors still demonstrate cognitive sequelae 3 years after the incident (Patel et al., 2003). Particularly long-term deficits in attention, memory and executive functions are common in stroke patients. Importantly, evidence suggests that specific neurofeedback protocols have largest effects on specific cognitive functions. For instance, Theta/Beta neurofeedback has mainly proven successful in improving attention and executive functions (Monastra et al., 2002; Fox et al., 2005; Duric et al., 2012), while SMR neurofeedback has been consistently found to improve memory functions (Vernon et al., 2003; Hoedlmoser et al., 2008; Hofer et al., 2014; Schabus et al., 2014; Kober et al., 2015b). Thus, neurofeedback training protocols can be chosen according to the patient’s cognitive deficits. In the present study, we chose to train a 74-years-old stroke patient with memory impairments with SMR neurofeedback to investigate training feasibility and the effects of the training on cognition and electrophysiological parameters. We chose to train the selected patients due to his very specific cognitive deficits that comprised memory impairments but no deficits of attention or executive functions. In a previous study, we found that patients with heterogeneous lesion locations could reach control over their brain activity during SMR-based and Upper Alpha-based neurofeedback (Kober et al., 2015a). Therefore, in the present study we chose to base our patient selection on the cognitive deficits observed rather than on lesion locations.

Hitherto, there is still a lack of studies assessing the efficiency and feasibility of neurofeedback protocols in stroke patients. In two studies reported by Doppelmayr et al. (2007), inconsistent results were observed regarding the efficiency of alpha and theta-based neurofeedback in stroke patients. While alpha neurofeedback proved superior to a control treatment in the first study, this was not replicated in a second study investigating alpha and theta neurofeedback. In this second study, neither alpha neurofeedback nor theta was more efficient than a control treatment. On the other hand, in a range of case studies, positive effects of neurofeedback on cognitive performance in stroke patients were reported (Rozelle and Budzynski, 1995; Bearden et al., 2003; Cannon et al., 2010). Still, these studies lack healthy control groups to assess whether stroke patients can benefit as much from the training as healthy persons. Therefore, in the present study we included an elderly control sample for comparison. Single-case control approaches allow the comparison of the patient’s improvement with the improvement observed in the healthy elderly (Crawford and Garthwaite, 2002, 2004). In a recent systematic study on stroke patients, Kober et al. (2015a) observed improvements in memory functions after SMR-based and Upper Alpha-based neurofeedback training. Effects were stronger than the effects of traditional cognitive training in a control group of stroke patients. While this study provided promising evidence that neurofeedback might be used as an effective tool for cognitive rehabilitation in stroke patients, the neuronal basis of the observed behavioral improvements remained unexplored. Thus, in the present study we set out to investigate electrophysiological parameters in detail in a stroke patients and a healthy elderly control group before and after neurofeedback training. Of note, we selected elderly participants as controls, as there is evidence that electrophysiological brain activity changes across the life span (Polich, 1997; Klimesch, 1999; Babiloni et al., 2006; Cummins and Finnigan, 2007; Rossini et al., 2007), which might also affect the functionality of neurofeedback paradigms. As neurofeedback studies in older persons are still scarce, an additional aim of the present study was the assessment of the efficiency of SMR neurofeedback training in the elderly sample. It has been demonstrated that across the lifespan, cognitive decline is accompanied by a broad array of changes in brain activation and structure (Hedden and Gabrieli, 2004; Rabbitt et al., 2007; Finnigan and Robertson, 2011). Based on such observations, one may assume that self-regulation of brain activity might be an efficient method to counteract age-related cognitive declines in older persons.

## Materials and Methods

### Participants

#### Stroke Patient UG20

UG20 (age 74, male) had suffered a stroke (ICD-10 diagnosis I63.1) due to basilar artery thrombosis (ICD-10 diagnosis I65.1) with lesions in right cerebellum, bilateral hippocampus, right mesencephalon, left occipital lobe, bilateral splenium 4.5 months before start of SMR training. At the time of stroke onset, UG20 was retired after working for several years as department head of an inventory department. He had completed 12 years of education. After the stroke, UG20 stayed in neurorehabilitation for 3 months, then he was released back home. Since his stroke, UG20 has suffered from memory deficits (amnestic syndrome), while his score of 29 in the mini-mental state examination (MMSE, Folstein et al., 1975) did not indicate general cognitive deficits. UG20 had normal corrected vision in the whole visual field (no anopsia), intact hearing abilities and showed signs of slight dysarthria. He did not suffer from hemiparesis but showed a subtle motor restriction of the right foot and a slight unsteadiness in walking. UG20 was administered a detailed neuropsychological assessment before start of the training (pre-assessment) to ensure the selection of an adequate neurofeedback training for him. This neuropsychological test battery was repeated after the last neurofeedback training session (post-assessment). The results of the pre-assessment (see **Figure 4**) indicated that UG20 mainly suffered from short- and long-term memory deficits, while other cognitive functions (alertness, cognitive flexibility, divided attention, inhibition) remained unaffected. Thus, we decided to apply SMR neurofeedback training in UG20, as this training has proven to be specifically effective in improving memory performance. Neurofeedback was conducted for 10 sessions in the course of 3 weeks at UG20s home. During neurofeedback training, UG20 did not receive medications that affected vigilance or attention and did not take part in other forms of cognitive training. UG20 received an expense allowance of 7 Euro/hour. UG20 provided written informed consent before participation and consented to publication of his data.

#### Elderly Control Sample

As a reference for the evaluation of training-related changes in cognitive performance and EEG signal in UG20, we trained a control sample of *N* = 10 healthy elderly participants (*mean* age: 70.6, *SE* = 2.36, range: 60–84; see **Table 1** for demographic data of UG20 and the control group). Healthy participants were recruited from the general population, by public bulletins and newspaper announcements and gave their written informed consent before participation. Exclusion criteria were any current or previous psychiatric or neurological disorders and history of severe head injuries. Controls received an expense allowance of 7 Euro/hour. The ethics committee of the University of Graz, Austria, approved all aspects of the present study (reference numbers GZ. 39/21/63 ex 2011/12 and GZ. 39/22/63 ex 2011/12) and the study was in line with the code of ethics of the World Medical Association, Declaration of Helsinki. Participants provided written informed consent before participation.

**Table 1:** Demographic data of stroke patient UG20 and the healthy control group.

	UG20	Control Group


Age	74	*Mean* 70.6 (*SE* = 2.36)


Gender	Male	Five females, five males


Handedness	Right	Nine right, one both


Years of education	12	*Mean* 10.2 (*SE* = 0.63)




### SMR-Based Neurofeedback Training

Electroencephalography signals for SMR training were recorded at channel Cz, digitized at 256 Hz and filtered with a 0.5 Hz high-pass and a 60 Hz low-pass filter. A 10-channel system (NeXus-10 MKII, Mind Media BV) and a g.USBamp 16-channel standard amplifier (g.tec, Graz, Austria) were used for data recording. The ground electrode was located at the right mastoid, the reference at the left mastoid. Vertical electrooculogram (EOG) was recorded by two electrodes on top and below the left eye (bipolar montage). Each participant performed 10 training sessions (approximately 50 min each) on different days. Sessions were conducted 2–5 times a week and consisted of 10 min of preparation, a 3-min baseline trial in which the participants saw moving bars while instructed to relax themselves without trying to control the bars voluntarily, and six 3-min feedback runs. In these runs, participants were instructed to increase SMR power while reducing electromyography (EMG) and EOG artifacts. Three bars were presented on a computer screen: the height of the bar in the middle reflected real-time SMR power (12–15 Hz, band power), while the height of the left bar reflected eye-blink artifacts (4–7 Hz, EEG band power) and the height of the right bar EMG artifact power (EEG band power between 21 and 35 Hz which indicates movements and other high-frequency disturbances). The data recorded in the baseline trial was used to calculate individual thresholds for the three bars. For the artifact bars, thresholds were kept constant over all neurofeedback runs (baseline *mean* + 1 *SD*), while the threshold of SMR power was adapted automatically after each run on the basis of the previous run (*mean*). When the SMR band power exceeded the predefined threshold, the color of the bar changed from red to green. When participants were able to move the SMR bar above its threshold while keeping the artifact bars below their thresholds for more than 250 ms, they were rewarded by getting points shown at the bottom of the screen. Participants were not given any instruction of how to control the feedback bars but were only told to stay mentally focused and physically relaxed.

For neurofeedback data analysis, SMR (12–15 Hz) band power was extracted by means of a procedure provided by the Vision Analyzer software (complex demodulation procedure, Brain Products GmbH, Munich, Germany). The resulting power values were averaged over all artifact free segments of each training run. To assess training effects, SMR power was averaged per run of training across all different sessions. SMR power values were z-transformed to ensure comparability across sessions and subjects. To analyze more closely the time course of SMR power over the training runs averaged over all sessions, we conducted linear regression analyses (predictor variable = run; dependent variable = SMR power). As during successful neurofeedback, a within-session increase of the feedback frequency power is expected, the slope of the regression line was used as an indicator of neurofeedback performance (see Zoefel et al., 2011; Escolano et al., 2012; Witte et al., 2013).

### Pre- and Post-assessment

#### Behavioral Assessment

**FIGURE 1:**
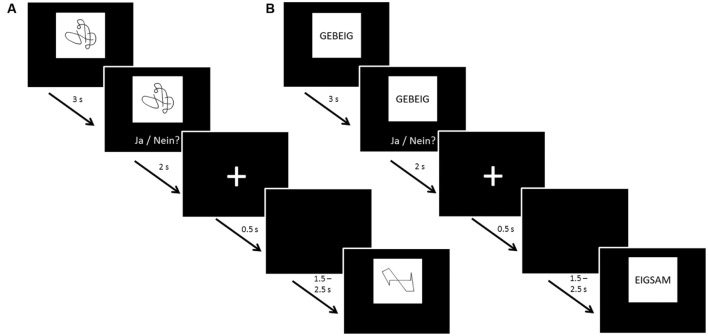
**Task procedure of the **(A)** non-verbal learning task (NVLT) and **(B)** verbal learning task (VLT).** After 3 s of stimulus presentation, participants were shown the stimulus and the question “Ja/Nein?” [German for “Yes/No”] for 2 s, asking them to indicate by button press whether they had seen the stimulus before. Then, a fixation cross was shown for 0.5 s. Before the next stimulus presentation, a black screen was shown for a jittered duration of 1.5–2.5 s to avoid expectation effects of the participants.

The same protocol was followed for UG20 and the healthy control sample: before and after neurofeedback training, 60-channel EEG measurements were carried out while participants were presented with a non-verbal (NVLT) and a verbal (VLT) memory task adapted from Sturm and Willmes (1999). Both tasks were divided into eight blocks consisting of 20 stimuli each. Eight of these 20 stimuli appeared in each of the blocks (repetition items), while the other 12 stimuli were shown only once each. Stimuli were presented for 3 s each, then participants had 2 s to react by button press. Between stimuli a fixation cross was displayed for 500 ms, followed by a black screen shown for a duration of 1.5–2.5 s (jittered; see **Figure 1** for a depiction of task procedure). In the NVLT, participants saw geometric or irregular shapes consisting of black lines on a white rectangle presented on a black background screen. After seeing each shape, participants had to indicate by button press whether they had seen this figure before or not. The VLT followed the same procedure, but instead of figures, pseudo-words (neologisms) were used as stimuli. Neologisms were presented in black letters (2 cm font size) on a white rectangle over a black background screen.

UG20 additionally performed a comprehensive neuropsychological test battery before and after neurofeedback training to evaluate the cognitive profile of the stroke patient in more detail. This test battery included standardized neuropsychological tests to assess attention [Subtest Alertness of the Test of Attentional Performance (TAP) test battery, Zimmermann and Fimm, 2009], divided attention (Subtest Divided Attention of the TAP test battery, Zimmermann and Fimm, 2009), inhibition (Subtest Go/NoGo of the TAP test battery, Zimmermann and Fimm, 2009), cognitive flexibility (Subtest Flexibility of the TAP test battery, Zimmermann and Fimm, 2009), verbal long-term memory (California Verbal Learning Test CVLT, Niemann et al., 2008; Visual and Verbal Memory Test VVM2 subscale “construction 2,” Schelling and Schächtele, 2001), non-verbal long-term memory (VVM2 subscale “city map 2,” Schelling and Schächtele, 2001), verbal short-term memory (VVM2 subscale “construction 1,” Schelling and Schächtele, 2001; CVLT List A Trial 1 and List B, Niemann et al., 2008; Wechsler Memory Scale WMS-R Digit Span test forward task, Härting and Wechsler, 2000), non-verbal short-term memory (VVM2 subscale “city map 1,” Schelling and Schächtele, 2001); Corsi Block Tapping Test (CBTT forward task, Schellig, 2011) and working memory (CBTT backward task, Schellig, 2011); Digit Span test backward task of the (WMS-R, Härting and Wechsler, 2000). Parallel forms of the memory tests were used to avoid learning effects.

#### Electrophysiological Parameters

During the pre- and post-assessments, multichannel EEG was recorded from 60 electrode sites according to the International 10–20 EEG placement system with a sampling frequency of 500 Hz and band-pass filter (0.01–100 Hz). Multichannel EEG amplifiers (BrainAmp DC by Brain Products, Munich, Germany and g.USBamp by g.tec, Graz, Austria) were used (reference: linked-ear). Furthermore, horizontal and vertical EOG was recorded using an electrode placed at the nasion and two electrodes placed at the left and right temples, as in previous studies (Schlögl et al., 2007). Impedances were kept below 5 kOhms for EEG and below 10 kOhms for EOG recordings.

Data was preprocessed and analyzed with Brain Vision Analyzer software (Brain Products GmbH, Munich, Germany). The signal was filtered offline with a 0.1 Hz high-pass and 100 Hz low-pass. EOG artifact correction was carried out using independent component analysis (ICA) ocular correction. Other EEG artifacts (e.g., muscle artifacts) were automatically rejected when one of the following criteria was fulfilled: >50 μV voltage step per sampling point, absolute voltage value exceeding ±120 μV.

For the verbal and non-verbal learning task, we analyzed ERPs in the EEG after presentation of target stimuli participants responded correctly to. Based on previous results of our group (Kober et al., 2015b), we analyzed the ERPs P3 and N1. In a first step, the period from 200 ms prior to 1000 ms following stimuli onset, relative to a 200 ms pre-stimulus baseline was extracted. For N1, signal over electrode FCz was analyzed, while for P3 we extracted the signal from Pz. Then, the mean area ERP amplitude for the healthy group was obtained by averaging the amplitude of the signal for latency windows chosen depending on the ERP characteristics in each task: VLT: N1 = 110–170, P3 = 500–750 ms; NVLT: N1 = 110–170, P3 = 450–650 ms after stimulus onset. As UG20 showed a slightly later N1, for him a latency window of 180–250 was used in both tasks. We calculated difference values by subtracting the mean area ERP amplitude values during the post-measurement from the mean area ERP amplitude values during the pre-measurement. For N1, negative values indicate larger N1 amplitudes during post- compared to pre-measurement. For P3, positive values indicate larger P3 amplitudes during post- compared to pre-measurement. For both N1 and P3, a minimum number of 15 trials was used for averaging.

Additionally, coherence analyses were applied to assess connectivity between motor areas and parietal brain areas during the learning tasks. EEG coherence is a measure of the degree of synchronous electrical activity in different brain areas over time. Coherence was assessed during the baseline intervals from 500 ms prior to stimulus onset. In a first step, for each segment, Fast Fourier Transformation (FFT, maximum resolution of ∼0.98 Hz, 10% Hanning window including variance correction) was applied to calculate EEG power spectra. Coherence *r* in the SMR band 12–15 Hz was then calculated between the channels Cz and CPz, Cz and Pz, and Cz and POz (for a more detailed description of the procedure see Kober et al., 2015b). The resulting *r*-value is a generalization of the Pearson product correlation coefficient to frequency domain variables and ranges from 0 (no correlation in frequency) to 1 (ideal constant correlation). Coherence values were Fisher’s *z*-transformed to normalize the distributions. For reporting, means were inverse transformed.

#### Statistical Analysis of Pre–post Differences

For the healthy participants, differences in scores of the learning tasks, ERP amplitudes and coherence were assessed by means of paired-sample *t*-test (evaluated at the two-sided significance level of α = 5%). To ascertain the degree of homogeneity in the control group and to investigate the robustness of training results in the sample, non-parametric bootstrapping methods were employed. Pre–post comparisons were bootstrapped 10000 times (*n* = 10 with replacement). The median *p*-value generated by bootstrapping will be reported complementarily. To assess the response of UG20 to training, the differences in his test scores were compared to the pre–post difference of healthy controls applying single-case analysis based on methods by Crawford and Garthwaite, 2002). These methods enable the assessment of the probability that test scores and test score discrepancies of a patient and a modest-sized control sample belong to the same distribution (Crawford and Garthwaite, 2002).

Moreover, to analyze clinical relevance of pre–post changes of the neuropsychological test battery in UG20, critical difference analysis (Huber, 1973) were performed. To identify significant improvement or decline, the critical difference of the relevant test parameter was compared with the test score difference obtained during the post-assessment minus the pre-assessment. The difference between pre- and post-assessment shown by UG20 was considered significant when it was larger than the critical difference, which can be detected by each test and only occurs in the population with a probability lower than α < 10%.

## Results

### Neurofeedback Performance

**FIGURE 2:**
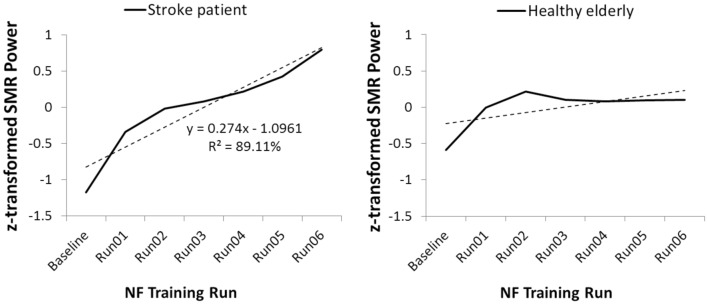
**Time course of sensorimotor rhythm (SMR) power over the neurofeedback training runs, averaged over all 10 NF training sessions, in stroke patient UG20 and in the healthy elderly group**.

**Figure 2**, left side, illustrates the time course of SMR power over the training runs for the stroke patient UG20. A clear increase in SMR power across training runs can be observed in UG20, which was also reflected in the regression slope resulting from a linear regression analysis.

As shown in **Figure 2**, right side, healthy participants were also able to increase SMR power over the training runs, as their linear regression slopes were significantly larger than 0 in a two-sided *t*-test [*t*(9) = -2.04, *p* < 0.05, *d* = -1.29]. The results of this regression analysis indicated that participants showed consistent linear increase in SMR power over the training runs (averaged over all 10 training sessions). When analyzing the time course of SMR power over the training runs separately for each participant of the SMR group, 8 out of 10 participants (i.e., 80%) showed a positive gradient of the learning curve. Of the two remaining participants, one showed a flat learning curve gradient and one a negative gradient. A single-case analysis (Crawford and Garthwaite, 2004) indicated that the patient’s regression slope did not differ significantly from the healthy participants’ slopes [*t*(9) = 1.93, *p* = 0.09, *d* = 1.22], indicating that his ability to alter SMR power was at least as good but not statistically different from that of healthy controls. The increase in SMR power observed in UG20 across training runs indicated successful training of SMR in the stroke patient.

### Behavioral Results

**FIGURE 3:**
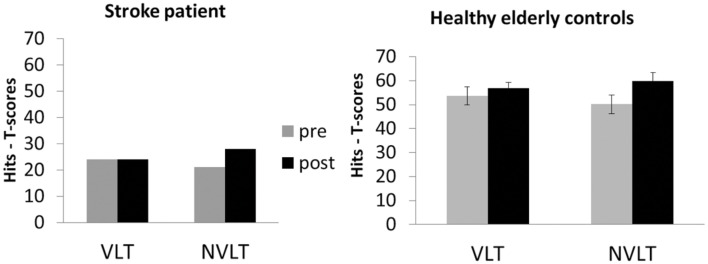
**Means and standard errors of the performance in the verbal learning task (VLT) and the non-verbal learning task (NVLT) for UG20 and the control group before and after NF training**.

After neurofeedback training, UG20 improved in the non-verbal task, while performance in the verbal learning task remained unaltered after training (see left side of **Figure 3**). Healthy participants showed improvements in the non-verbal learning task after training as well, which were significant in a *t*-test [see **Figure 3**; *pre*–*post*: *t*(9) = -2.03, *p* < 0.05, *d* = -1.28, *p_bootstrapping_* = 0.035]. In the verbal learning task, no change in performance was observed in the controls [*pre*–*post*: *t*(9) = -0.74, n.s., *d* = -0.47, *p_bootstrapping_* = 0.30]. To further assess the improvement observed in UG20, the pre–post difference in his test scores was compared to the pre–post difference of healthy controls applying single-case analysis according to the approach of Crawford and Garthwaite (2002). The patient’s and the controls’ values did not differ significantly [*t*(9) = -0.127, *p* = 0.451, *d* = -0.08], indicating that UG20 improved at least as much as the controls in the NVLT after the training.

**FIGURE 4:**
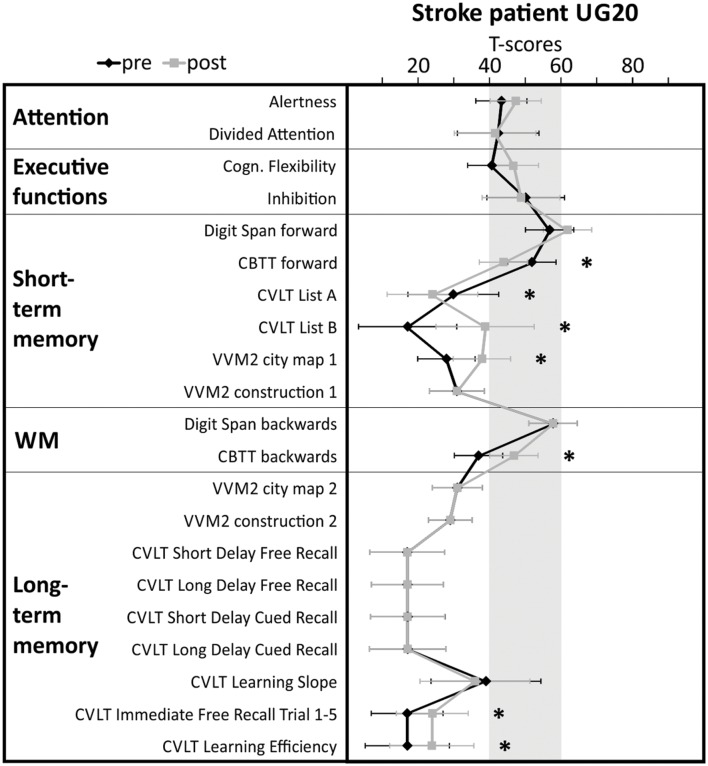
**Results of the detailed cognitive assessment carried out in stroke patient UG20 before and after neurofeedback training.** Shaded area indicates norm scores. Test performance is expressed in T-scores with population mean *M* = 50 and standard deviation *SD* = 10. Neuropsychological test scores and confidence intervals for measurements of alertness, divided attention, cognitive flexibility and inhibition scores were derived from tasks of the Testbatterie zur Aufmerksamkeitsprüfung (Zimmermann and Fimm, 2009). STM, short-term memory; LTM, long-term memory; CBTT, Corsi Block Tapping Test (Schellig, 2011); VVM2, Visueller und Verbaler Merkfähigkeitstest 2 (Schelling and Schächtele, 2001); CVLT, California Verbal Learning test (Niemann et al., 2008). Significant differences between pre- and post-test (critical difference analysis on the group level, Huber, 1973) are marked with asterisks (^∗^significant).

In the neuropsychological test battery assessed before and after neurofeedback training, stroke patient UG20 showed significant performance improvements in non-verbal short-term memory (VVM2 subscale “city map 1”) and working memory (CBTT backward task) tasks when comparing the pre- and post-assessment. The performance in several scales of the CVLT assessing verbal short- and long-term memory performance also improved (List B, Immediate Free Recall, Learning Efficiency). In the forward task of the CBTT and List A of the CVLT assessing short-term memory performance, UG20 showed a decreased performance after neurofeedback training compared to the pre-assessment. In sum, UG20 showed significant improvements in memory tasks, whereas attentional and executive functions did not change when comparing the results of the pre- and post-assessment (**Figure 4**).

### Electrophysiological Results

#### Event-Related Potentials

**FIGURE 5:**
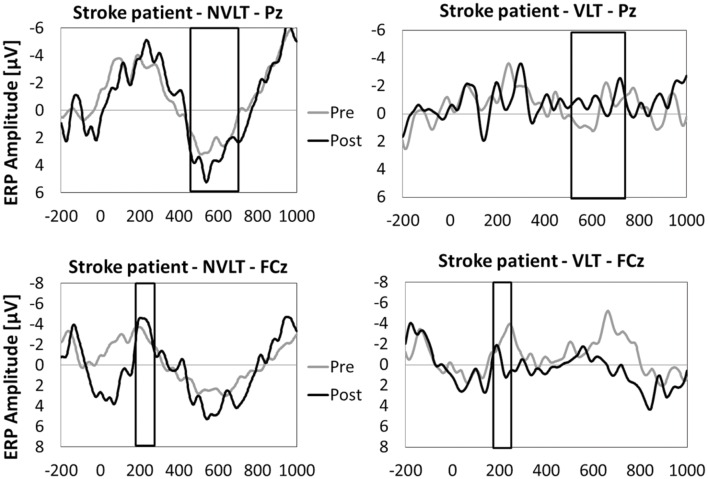
**Grand average event-related potentials (FCz and Pz) during the encoding phase of the non-verbal learning task (NVLT) and the verbal learning task (VLT), for the pre- and post-measurement of the healthy elderly group.** The analyzed latency windows are marked with rectangles.

**FIGURE 6:**
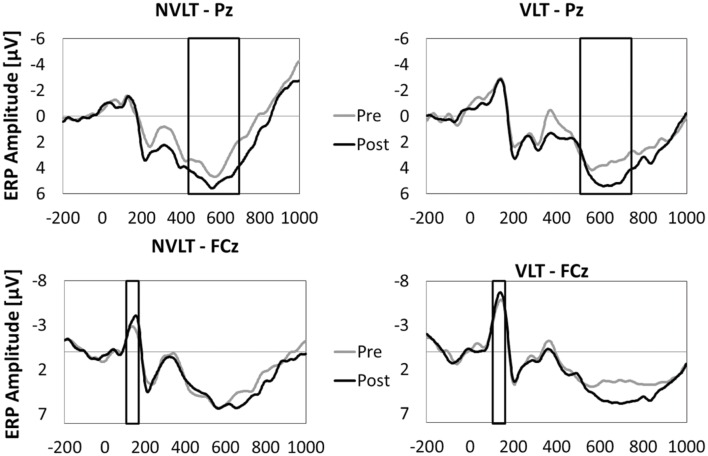
**Means of ERP amplitudes (FCz and Pz) during the encoding phase of the non-verbal learning task (NVLT) and the verbal learning task (VLT), for the pre- and post-measurement of stroke patient UG20.** The analyzed latency windows are marked with rectangles.

In **Figures 5** and **6**, the grand average ERPs from Pz are illustrated for UG20 and the healthy elderly group during pre- and post-measurement. For UG20, an alteration in P3 amplitude was observed in the non-verbal learning task (see **Figure 6**): P3 amplitude was enhanced post-training compared to pre-training. N1 amplitude in the non-verbal task was slightly increased in UG20 after training as well. In the verbal learning task, no clear pre–post differences in P3 or N1 amplitudes could be observed in UG20 (see **Figure 6**).

The healthy group showed a tendency for a higher P3 amplitude in the post-compared to the pre-measurement during stimulus processing of the non-verbal task [*t*(9) = 1.91, *p* = 0.08, *d* = 1.21, *p_bootstrapping_* = 0.078; see **Figure 5**]. When comparing the N1 amplitude between pre- and post-test, no differences were found [*t*(9) = 0.82, n.s., *d* = 0.51, *p_bootstrapping_* = 0.36]. In the verbal task, there were neither pre–post differences in P3 amplitudes [*t*(9) = 1.12, n.s., *d* = 0.71, *p_bootstrapping_* = 0.27] nor in N1 amplitudes [*t*(9) = 0.66, n.s., *d* = 0.42, *p_bootstrapping_* = 0.41].

A single-case-analysis (Crawford and Garthwaite, 2002) comparing the post-pre difference in P3 amplitudes during the non-verbal task between the patient and healthy participants indicated no significant difference [*t*(9) = -0.005, *p* = 0.498, *d* = -0.003] between these values, thus indicating a tendency of amplitude increase of similar extent as observed in the controls. Also, there was no significant difference between the patient’s and the healthy participants’ change in N1 according to single-case analysis based on Crawford and Garthwaite (2002; *t*(9) = -0.016, *p* = 0.494, *d* = -0.016), indicating no significant change in N1 in the patient.

#### Coherence

**FIGURE 7:**
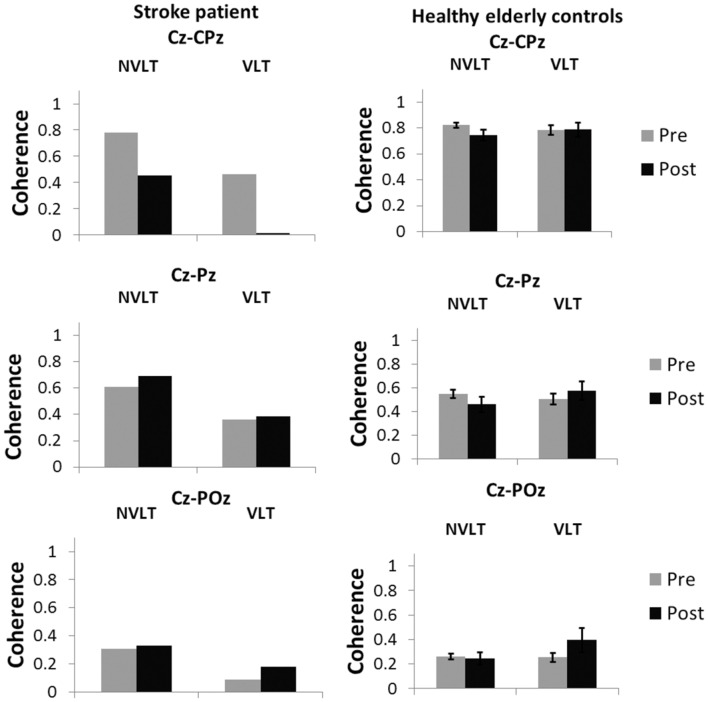
**Coherence values (*M* and SE) in the SMR band (12–15 Hz) for the electrode pairs Cz-CPz, Cz-Pz and Cz-Poz for the healthy elderly group and stroke patient UG20**.

Coherence analyses for the non-verbal task indicated a decrease in coherence between electrodes Cz and CPz during the non-verbal task for UG20. In the verbal learning task, coherence decreased as well (see **Figure 7**).

For healthy controls, coherence during the non-verbal learning task between electrodes Cz and CPz showed a marginally significant decrease after NF compared to pre-training values [*t*(9) = 1.92, *p* = 0.09, *d* = 1.21, *p_bootstrapping_* = 0.06]. For the other two electrode pairs, coherence did not differ significantly after training when compared to before training [Cz-Pz: *t*(9) = 1.75, *p* = 0.11, *d* = 1.11, Cz-Poz: *t*(9) = 0.45, *p* = 0.66, *d* = 0.28, *p_bootstrapping_* = 0.11]. For the verbal task, no differences in coherence were observed [Cz-CPz: *t*(9) = 0.06, *p* = 0.95, *d* = 0.04, *p_bootstrapping_* = 0.50, Cz-Pz: *t*(9) = 0.83, *p* = 0.43, *d* = 0.52, *p_bootstrapping_* = 0.35, Cz-Poz: *t*(9) = 1.48, *p* = 0.17, *d* = 0.94, *p_bootstrapping_* = 0.16].

Single-case analysis (Crawford and Garthwaite, 2002) for the non-verbal learning task showed that there was a significant difference between the patient’s change in coherence between Cz and CPz and the healthy controls’ [*t*(9) = -1.871, *p* = 0.047, *d* = -1.18]: the decrease in UG20s coherence values was larger than the decrease in the healthy elderly. In the verbal learning task, according to single-case analysis, the decrease in coherence was also significantly larger in UG20 than in healthy controls [*t*(9) = -2.080, *p* = 0.03, *d* = -1.32]. There were no significant differences between pre–post coherence changes of UG20 and the controls in the other two electrode pairs and in the verbal learning task (all *p*s > 0.1, see **Figure 7**).

## Discussion

The present study investigated the applicability of SMR-based neurofeedback training in one stroke patient (UG20) and a healthy elderly control sample and assessed behavioral and electrophysiological effects of the training. Results indicated that participants learned to increase SMR voluntarily during neurofeedback. After 10 sessions of SMR up-training, UG20 and the control group showed behavioral improvements in a non-verbal learning memory task, an increase in P3 amplitudes and a decrease in electrophysiological coherence during this task. Single-case Crawford analyses indicated that the changes observed in UG20 were as large as the changes in the age-matched controls, suggesting that UG20 benefited as much from the training as the control group. These results will be discussed in more detail below.

In a first step, we assessed whether UG20 was able to increase SMR activity voluntarily through neurofeedback training. Regression analysis showed that during the neurofeedback training sessions, UG20s SMR activity increased linearly, indicating that he was able to voluntarily increase SMR activity. The control group also showed consistent increases in SMR power over the training runs averaged, indicating successful up-training of SMR activity. This finding is consistent with previous studies demonstrating the applicability of slow cortical potentials-, alpha- and theta-based neurofeedback paradigms in healthy older persons (Kotchoubey et al., 2000; Angelakis et al., 2007; Wang and Hsieh, 2013). A single-case Crawford analysis (Crawford and Garthwaite, 2002, 2004) comparing the regression slope of UG20 and the control group showed that there was no difference in learning ability between UG20 and the controls. These results support previous case studies showing that stroke patients are able to control their brain activity during neurofeedback training (Bearden et al., 2003; Cannon et al., 2010) and extend the evidence by showing that UG20 was equally able to up-regulate SMR activity as age-matched healthy controls.

Before and after neurofeedback training, UG20 was confronted with a verbal learning task and a non-verbal learning task. UG20 showed an improvement in the non-verbal learning task after neurofeedback training. The control group’s results mirrored those of UG20, as they showed a significant improvement of performance in the non-verbal learning task as well. A single-case Crawford analysis indicated that UG20 improved his non-verbal memory performance as much after the training as the healthy elderly group. These results are well in line with studies demonstrating improved memory abilities after SMR neurofeedback training (Vernon et al., 2003; Hoedlmoser et al., 2008; Schabus et al., 2014; Kober et al., 2015b). Further, they support results of a previous study of our group showing improved memory performance after SMR neurofeedback in healthy young participants (Kober et al., 2015b). Based on these findings, we suggested that up-regulation of SMR leads to a reduction of sensorimotor interferences. This inhibition of sensorimotor interferences enhances stimulus processing and thereby leads to improved memory performance (Kober et al., 2015b). Thus, in our present study we found that these mechanisms could also be triggered in a stroke patient suffering from broad memory deficits as well as in healthy elderly persons not affected by memory deficits.

While UG20 as well as healthy elderly controls showed improvements in their non-verbal memory performance after SMR based neurofeedback training, no change in performance was observed in the verbal learning task. This finding contrasts with our previous study (Kober et al., 2015b), where we observed behavioral improvements in a verbal memory task after training. One reason for this discrepancy could lie in the nature of the applied tasks: while in our previous study, participants improved in a free-recall task of verbal material presented in a story-like format (subtest “construction” of the Visual and Verbal Memory Test; Visueller und verbaler Merkfähigkeitstest2 – Schelling and Schächtele, 2001), in the present study a recognition task with pseudo-words was used. As suggested in Kober et al. (2015b), we suppose that SMR up-training leads to an improved suppression of sensorimotor interferences, which facilitates the formation of deep semantic associations and therefore enhances later retrieval. The verbal task used in the present study, however, does not allow the formation of deep semantic associations as the items that should be memorized were pseudo-words. This might be a reason why this task was not suitable to capture changes induced by SMR neurofeedback training. Still, the question arises why participants then improved in the non-verbal learning task in our study. In contrast to the verbal learning task with pseudo-words, the non-verbal learning task contains partially associative figures (e.g., a symbol resembling a house) that could allow for the formation of semantic associations. Another possible explanation for our findings is that non-verbal memory might be affected by aging earlier than verbal memory (Jenkins et al., 2000; Murre et al., 2013). This might lead to the non-verbal learning task being more sensitive to performance improvements induced by SMR neurofeedback. The results of the neuropsychological test battery, which was performed by UG20 before and after neurofeedback training, support this assumption. UG20 improved his performance in the non-verbal short-term memory task of the VVM2 (subscale “city map 1”), but not in the verbal subtest “construction” of the VVM2 when comparing pre- and post-assessment. However, the healthy elderly group did not show performance differences between the verbal and non-verbal learning task during the pre-assessment, which is in contrast to the hypothesis that non-verbal memory might be affected by aging earlier than verbal memory. Further studies applying more varieties of memory tasks to healthy elderly controls are necessary to elucidate the specificity of SMR training effects on verbal and non-verbal memory functions and on the formation of semantic associations.

The results of the neuropsychological test battery suggest that SMR neurofeedback training had mainly effects on memory functions in stroke patient UG20. Before the start of the neurofeedback training, UG20 showed severe memory deficits (T-scores < 40). After SMR neurofeedback, UG20 showed a higher performance in different memory functions compared to the pre-assessment. For instance, working memory performance as assessed with the CBTT backward task was below average during the pre-assessment. After SMR neurofeedback training, performance in the CBTT backward task was in a normal range. Short-term memory performance (VVM2 “city map 1” and CVLT List B) was also far below average before the start of the neurofeedback training. During the post-assessment, performance in these short-term memory tasks was only slightly lower than the normal range. Attentional and executive functions did not change due to SMR neurofeedback training. This result is in line with previous findings in healthy people that indicated that SMR neurofeedback training leads to memory improvements (Vernon et al., 2003; Hoedlmoser et al., 2008; Gruzelier, 2014; Hofer et al., 2014; Schabus et al., 2014; Kober et al., 2015b). Of note, UG20 did not participate in other cognitive training and did not take medication while taking part in our study. Thus, we can exclude that the improvements we observed are due to these factors. However, we cannot entirely exclude the influence of other factors, for instance socialization on the changes in memory function. Some small performance declines could be observed in UG20 in two scales of the short-term memory assessment (CBTT forward task and CVLT List A). The reduced performance in these two tasks after neurofeedback training compared to the pre-assessment might be caused by reduced motivation or inattention, since an improvement in working memory performance (CBTT backward task) would not be possible if short-term memory performance in UG20 had declined seriously. For successful short-term memory performance, items have to be memorized and retrieved after a short delay. For successful working memory performance, items have to be memorized, retrieved and mentally transformed. Working memory refers to the structures and processes used for temporarily storing and manipulating information. Hence, an intact short-term memory can be regarded as a prerequisite for successful working memory performance.

While completing the non-verbal and verbal learning tasks, our participants underwent 60-channel EEG measurements enabling us to assess ERPs during stimulus processing. In line with the behavioral results, in UG20 changes in EEG parameters after neurofeedback training were only apparent in the non-verbal learning task: in this task, he showed higher P3 amplitudes after training compared to the pre-measurements. N1 amplitude was also slightly higher in UG20 after training than before. In the healthy elderly group, a similar pattern was observed: analyses of ERPs showed a tendency for higher P3 amplitudes in the post-compared to the pre-measurements during presentation of correctly identified target stimuli. However, there was no change in N1 amplitude after training in the control group. Single-case analyses showed that the pre–post change in P3 amplitude in UG20 was not significantly different from the pre-post-change observed in healthy controls. Further, the change in N1 amplitude visible in UG20 was not larger than the change observed in the healthy elderly group. Thus, while neither UG20 nor the control group showed a reliable difference in N1 amplitude after training, they showed a tendential increase in P3 amplitude after training. The observed increase in P3 amplitude is consistent with previous results (Egner and Gruzelier, 2001; Kober et al., 2015b) and indicates more intensive stimulus processing after SMR up-training (Kok, 2001; Polich, 2007). In the context of memory tasks, increased P3 amplitudes are regarded as an indicator of more successful encoding, which facilitates later retrieval and recognition (Polich, 2007). Thus, this finding fits well with the behavioral improvements in the task apparent in UG20 and the control group. N1 is has also been related to cognitive processing, especially in attention tasks involving expectancy effects and in tasks assessing short-term memory (Golob and Starr, 2000; Herrmann and Knight, 2001; Correa et al., 2006; Fu et al., 2008). Therefore, we expected an enhancement of this component after training. The lack of a change in N1 in the control group could be due to large inter-individual variations in N1 amplitude observed in our study. Moreover, the comparatively small number of trials in our task might have contributed to this lack of an effect on N1 amplitude.

Furthermore, coherence analyses were carried out assessing electrophysiological coherence between the feedback region (electrode Cz) and parietal areas. In UG20, coherence between Cz and CPz decreased strongly after training compared to before and this change was apparent in both the verbal and the non-verbal learning task. This reduced coherence between motor and parietal areas indicates lower functional connectivity between these brain areas after training. Thus, activity in these regions was less synchronized after training, which could be a further indicator of a reduction of sensorimotor interferences achieved by SMR neurofeedback training. In the control group, coherence in the non-verbal learning task was marginally decreased after training compared to pre-training, and there were no changes in coherence in the verbal learning task. Single-case analyses indicated that electrophysiological coherence during the verbal and non-verbal learning task decreased more strongly in UG20 than in the healthy participants, further corroborating that SMR training was effective in UG20. Of note, compared to our previous results in healthy young participants (Kober et al., 2015b), the connectivity changes were less widespread in the stroke patient and the elderly persons as we could not observe changes in coherence between Cz and more posterior electrodes (POz, Pz).

The behavioral and electrophysiological results of our study partially replicate a previous study of our group on SMR neurofeedback in healthy young participants (Kober et al., 2015b). In this study, healthy young participants showed improved verbal memory, higher P3 and N1 amplitudes and decreased coherence between motor areas and parietal-occipital brain areas after SMR up-training. Based on these findings, we suggested that SMR up-training might lead to an improved shutting-down of sensorimotor interferences after training, which in turn results in enhanced stimulus processing (Kober et al., 2015b). In a recent study, we could show that stroke patients can benefit from SMR-based neurofeedback training, as they showed improved memory performance after training (Kober et al., 2015a). The present study set out to explore whether the observed memory improvements in stroke patients might also be related to the mechanism of shutting-down of sensorimotor interferences we proposed for younger participants (Kober et al., 2015b). As there is plenty of evidence showing that both resting-state EEG and task-related EEG activity change across the lifespan (Polich, 1997; Klimesch, 1999; Babiloni et al., 2006; Cummins and Finnigan, 2007; Rossini et al., 2007), in the present study we also set out to further investigate these processes in an elderly sample. Our results are in line with an assumed enhanced shutting-down of sensorimotor intereferences, as we observed a similar pattern of electrophysiological results as in Kober et al. (2015b): our ERP results provide evidence that up-regulation of SMR is related to enhanced stimulus processing of task relevant information. Furthermore, we observed a reduced coherence between motor areas (Cz) and more parietal areas which are more related to visual processing. As proposed by Sterman (1996), we assume that motor activity might disengage cortical visual processing areas, resulting in a compromising effect on information processing. Thus, we hypothesize that up-regulation of SMR activity is associated to a reduction of motor interferences and reduces the somatosensory information flow to motor areas. According to our interpretation, this “shutting down of sensorimotor interferences” in turn leads to an enhanced information processing (Kober et al., 2015b). Our results indicate that SMR up-training could enhance the suppression of sensorimotor interferences not only in healthy young, but also in elderly participants and in a stroke patient. These results are also in line with a previous study of our group, in which age was not a significant predictor of the ability to regulate SMR in a sample with a wide age-range (Reichert et al., 2015) and extend this finding by showing that SMR training has similar behavioral and electrophysiological effects in young and elderly participants and even in a stroke patient.

## Conclusion

In the present case-study, we could show that a stroke patient and healthy elderly controls were able to self-regulate their SMR activity during 10 sessions of SMR neurofeedback training. Moreover, observed behavioral improvements after training in a non-verbal learning task were accompanied by increased P3 amplitudes and a decreased coherence between motor and parietal areas during this task. These results offer support for the hypothesis that SMR neurofeedback enhances the inhibition of sensorimotor interferences, thereby facilitating stimulus processing and the formation of deep semantic associations. Furthermore, the improvements we observed in the UG20 were not smaller than those in the healthy controls, suggesting that neurofeedback might be a suitable tool for stroke patients and should be investigated further in future studies. Research on neurofeedback for stroke rehabilitation is of special importance as positive effects of traditional cognitive trainings in this domain remain disputed (das Nair and Lincoln, 2007; Hoffmann et al., 2010). Also, the technological developments in the last years have facilitated the implementation of neurofeedback training tremendously. In the future, even the independent realization of neurofeedback training at the homes of patients will be possible, as there are already sufficiently small amplifiers and easy-to-use headsets that can be set up by caregivers or patients themselves. Thus, neurofeedback provides an enormous potential for home-based rehabilitation that could add to the currently utilized traditional cognitive training programs.

## Author Contributions

JR: participated in the design of the study and data acquisition, analyzed, and interpreted data; and prepared the manuscript. SK: participated in the study design, analyzed data, and critically revised the manuscript. DS: acquired data, contributed to study design, carried out patient recruitment and the patient’s clinical assessment. PG: contributed to study coordination, patient recruitment, data collection, and medical care of the patient. GW: participated in the study design and coordination, performed statistical analysis and critically revised the manuscript. CN: participated in conception and supervision of the study, interpreted data and contributed to manuscript revision. All authors read and approved the final manuscript.

## Conflict of Interest Statement

The authors declare that the research was conducted in the absence of any commercial or financial relationships that could be construed as a potential conflict of interest.
